# No reduced serum serotonin levels in patients with post-acute sequelae of COVID-19

**DOI:** 10.1007/s15010-024-02397-5

**Published:** 2024-10-02

**Authors:** Philipp Mathé, Veronika Götz, Katarina Stete, Dietrich Walzer, Hanna Hilger, Stefanie Pfau, Maike Hofmann, Siegbert Rieg, Winfried V. Kern

**Affiliations:** Department of Medicine II, Medical Center, Faculty of Medicine, University of Freiburg, Hugstetter Str. 55, 79106 Freiburg, Germany

**Keywords:** Serum serotonin, SARS-CoV-2, Chronic fatigue syndrome, PASC, Biomarker

## Abstract

**Purpose:**

Approximately 10–20% of patients previously infected with SARS-CoV-2 experience post-acute sequelae of COVID-19 (PASC), presenting with fatigue and neurocognitive dysfunction along various other symptoms. Recent studies suggested a possible role of a virally induced decrease in peripheral serotonin concentration in the pathogenesis of PASC. We set out to verify this finding in an independent and well-defined cohort of PASC patients from our post-COVID-19 outpatient clinic.

**Methods:**

We performed a retrospective case–control study including 34 confirmed PASC patients and 14 healthy controls. Clinical assessment encompassed physician examination as well as questionnaire based evaluation. Eligibility required ongoing symptoms for at least 6 months post-PCR-confirmed infection, relevant fatigue (CFS ≥ 4), and no other medical conditions. Serum serotonin was determined by LC–MS/MS technique.

**Results:**

Serum serotonin levels in PASC patients did not significantly differ from healthy controls. Most subjects had normal serotonin levels, with no subnormal readings. Subgroup analyses showed no significant differences in serotonin levels based according to predominant fatigue type, high overall fatigue score or depression severity.

**Conclusion:**

We postulate that peripheral serotonin is no reliable biomarker for PASC and that it should not be used in routine diagnostic. Therapy of PASC with serotonin-reuptake inhibitors or tryptophane supplementation should not be based solely on the assumption of lowered serotonin levels.

**Supplementary Information:**

The online version contains supplementary material available at 10.1007/s15010-024-02397-5.

## Introduction

Approximately 10–20% of patients previously infected with SARS-CoV-2 suffer from post-acute sequelae of COVID-19 (PASC) [1], characterized by fatigue, neurocognitive dysfunction and various other complaints which may last for months and even years. Recent studies suggested a protective effect against PACS by the intake of selective serotonin reuptake inhibitors (SSRIs) during acute COVID-19 [2,3]. The pathogenesis of this process is not well understood. Two metabolomics studies suggested a possible role of virally induced decreases in peripheral serotonin concentrations in PASC [4,5]. Serotonin has been associated with modulatory effects on mood and anxiety, but also linked to altered sleep, cognition, and memory, with a circadian change of serotonin concentrations in peripheral blood [6]. We therefore investigated whether low serum serotonin levels might discriminate patients with long-lasting PASC from healthy controls and could serve as diagnostic biomarker.

## Methods

This was a retrospective case–control study of patients presenting with PASC at our post-COVID-19 outpatient clinic. Clinical assessments included a semi-structured interview and examination by a physician, neurocognitive testing with the Montreal Cognitive Assessment (MoCA), and validated questionnaires for fatigue (Chalder Fatigue Scale [CFS]) and depression (Patient Health Questionnaire-9 [PHQ-9]). Patients were included if they gave written consent, reported ongoing symptoms for at least 6 months with onset up to four weeks after PCR-confirmed SARS-CoV-2 infection, had relevant fatigue with a Chalder Fatigue Score ≥ 4 points (binary scale), had no other medical conditions explaining the symptoms as assessed by a trained physician, had thrombocyte concentrations between 150,000 and 350,000/µl, and did not receive any known interfering medication. Blood sampling of patients was performed routinely between 9 and 12 a.m. and for healthy controls between 9 and 2 p.m.

Analysis of serum serotonin levels was performed using LC–MS/MS technique [7], with limits for normal values ranging from 40 to 200 µg/L. Sample size calculation was based on the mean differences in plasma serotonin concentrations reported by Wong et al.^4^ and required at least 12 subjects per group (β = 0.8, α = 0.05). 14 adult healthy volunteers served as control. Statistical analysis compared serotonin levels between groups using an unpaired two-tailed Student’s *t*-test calculated with Graph Pad Prism 10. Ethical approval was granted by the ethics committee of the University of Freiburg (IRB-number 21-1254). All patients provided informed written consent. The study was registered in the German Database for Clinical Studies (DRKS00025392).

## Results

14 healthy controls and 34 PASC patients were included in this study. Demographic and clinical characteristics of patients are shown in Table 1. Briefly, patients had long-lasting PASC, with fatigue as the predominant complaint.

**Table 1:** Demographic and clinical characteristics of the 34 PASC patients included

Variable	N (%) or median (with interquartile range)	
	PASC patients	Healthy controls
Age, years	51 (39.5–57)	26.5 (24–37.25)
Sex		
*Female*	18/34 (53%)	7/14 (50%)
Time since initial COVID-19 infection episode (days)	410 (400–465)	280 (133–383)
Predominant self-reported symptoms		
*Fatigue*	31/34 (91%)	
*Difficulties of concentration/memory*	21/34 (62%)	
*Difficulties with breathing*	21/34 (62%)	
Questionnaire ratings		
*MoCA*	28 (24–29)	
*PHQ-9 depression score*	8 (5.5–12)	
*PHQ-9 symptom strength*	13 (6.75–17)	
*CFS scores*		
*Binary scale (0–11 points)*	9 (7–11)	
*Total (Likert scale) (0–33 points)*	23 (18.75–27)	
*Physical subscore (Questions 1–7)*	15 (11–19.25)	
*Mental subscore (Questions 8–11)*	8 (6–8.5)	

Abbreviations: *CFS* Chalder Fatigue Score,* MoCA* Montreal Cognitive Assessment-Test,* PASC* Post-Acute Sequelae of Covid-19,* PHQ* Patient-Health-Questionnaire

**Fig. 1:**
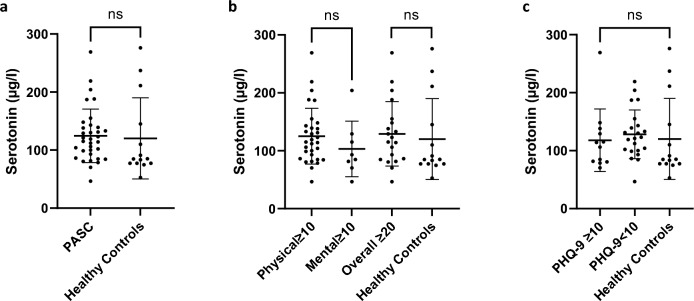
Mean serum serotonin concentrations in PASC patients and healthy control subjects. **a** Overall serotonin-levels in all PASC patients (n = 34) and healthy controls (n = 14). **b** Serotonin-levels in PASC patients according to high physical (n = 31) or mental fatigue scores (n = 8). **c** Serotonin-levels in PASC patients according to high (≥ 10) (n = 12) or low (< 10) (n = 22) scores in the 9-item depression scale (PHQ-9). Each dot represents one patient. Unpaired two-tailed Student’s t test was used for group comparisons. Statistical significance is indicated. Data are mean ± standard deviation. Abbreviations: *ns* = *not significant, PASC* post-acute sequelae of Covid-19, *PHQ*  patient health questionnaire

Serum serotonin levels in PASC patients (124.54 ± 46.02) were similar to those measured in healthy controls (120.24 ± 69.97, p = 0.8027) (Fig. 1a). Most patients and controls had normal range serotonin levels (31/34 [91%] versus 11/14 [79%]), and no subject had levels below the lower limit of normal. Subgroup analysis showed comparable serotonin levels for patients with predominant physical or mental fatigue (125.20 ± 48.07 versus 103.05 ± 48.05, p = 0.2527), or high overall fatigue scores (≥ 20) (129.22 ± 55.63) (Fig. 1b). Also, we observed no differences in serotonin levels between PASC patients with high or low PHQ-9 depression scores (117.93 ± 53.96 versus 128.14 ± 42.00, p = 0.5444) (Fig. 1c).

## Discussion

The present study could not confirm previously described differences in peripheral serotonin levels between PASC patients and healthy control subjects. We found no correlation of serotonin levels with the severity of PASC-associated fatigue or with the severity of depressive symptoms. Of note, there were no patients with subnormal serotonin levels. Sadlier and colleagues observed significantly lower serotonin levels in 20 PASC patients at two different follow-up times (compared to controls), together with other findings of a dysregulated metabolism of tryptophan. Significant differences were also observed in one of the three cohorts previously investigated by Wong and colleagues but these could not be replicated in the second cohort, while in a third cohort there was only a weak correlation of reduced serotonin and PASC-associated symptom number. Interestingly, previous cohort studies showed no correlation of plasma or serum levels of serotonin with depression after adjustments made for concurrent or recent antidepressant use.

Due to the limited number of study participants caused by a selection of only severe PASC cases, differences in age and insufficient correlation of peripheral and intracerebral serotonin concentrations, as well as relevant fluctuation in peripheral serotonin concentrations, a possible effect of altered serotonin levels on PASC cannot be excluded.

However, we postulate that peripheral serotonin is no reliable biomarker for PASC and that it should not be used in routine diagnostic.

Further studies are needed to further investigate a potential role of serotonin and related metabolites in the pathogenesis of PASC, especially with a focus on different body compartments and serotonin precursor metabolites. Therapy of PASC with serotonin-reuptake inhibitors or tryptophane supplementation (to prevent serotonin reduction) should be carefully evaluated and not be based solely on the assumption of lowered serotonin levels.Inclusion of a data availability statement is preferred for this journal. If applicable, please provide one.Data availability statement is included After "Funding"

## Supplementary Information

Below is the link to the electronic supplementary material.

Supplementary file1 (PPTX 141 KB)

## Data Availability

Data cannot be shared publicly as it contains some sensitive information. Anonymised data can be made available upon reasonable requests to the data access committee, via Nadine Mezger at the Freiburg University Hospital (nadine.mezger@uniklinik-freiburg.de).

## References

[1] Peter RS, Nieters A, Kräusslich HG, et al. Post-acute sequelae of covid-19 six to 12 months after infection: population based study. BMJ. 2022;379: e071050. 10.1136/bmj-2022-071050. 36229057 10.1136/bmj-2022-071050 PMC9557001

[2] Butzin-Dozier Z, Ji Y, Deshpande S, et al. SSRI Use During Acute COVID-19 Infection Associated with Lower Risk of Long COVID Among Patients with Depression. Published online February 6, 2024:2024.02.05.24302352. 10.1101/2024.02.05.24302352

[3] Sidky H, Hansen KA, Girvin AT, et al. Assessing the effect of selective serotonin reuptake inhibitors in the prevention of post-acute sequelae of COVID-19. Comput Struct Biotechnol J. 2024;24:115–25. 10.1016/j.csbj.2023.12.045. 38318198 10.1016/j.csbj.2023.12.045 PMC10839808

[4] Wong AC, Devason AS, Umana IC, et al. Serotonin reduction in post-acute sequelae of viral infection. Cell. 2023;186:4851-4867.e20. 10.1016/j.cell.2023.09.013. 37848036 10.1016/j.cell.2023.09.013 PMC11227373

[5] Sadlier C, Albrich WC, Neogi U, et al. Metabolic rewiring and serotonin depletion in patients with postacute sequelae of COVID-19. Allergy. 2022;77:1623–5. 10.1111/all.15253. 35150456 10.1111/all.15253 PMC9111264

[6] Rao ML, Gross G, Strebel B, et al. Circadian rhythm of tryptophan, serotonin, melatonin, and pituitary hormones in schizophrenia. Biol Psychiat. 1994;35:151–63. 10.1016/0006-3223(94)91147-9. 7909693 10.1016/0006-3223(94)91147-9

[7] Korse CM, Buning-Kager JCGM, Linders TC, et al. A serum and platelet-rich plasma serotonin assay using liquid chromatography tandem mass spectrometry for monitoring of neuroendocrine tumor patients. Clin Chim Acta. 2017;469:130–5. 10.1016/j.cca.2017.04.001. 28385629 10.1016/j.cca.2017.04.001

