# Supplementary figures and images for: No reduced serum serotonin levels in patients with post-acute sequelae of COVID-19
Source: Infection. 2024 Oct 2;53(1):463–6. doi: 10.1007/s15010-024-02397-5 (PMC11825522; doi:10.1007/s15010-024-02397-5)

## Slide 1
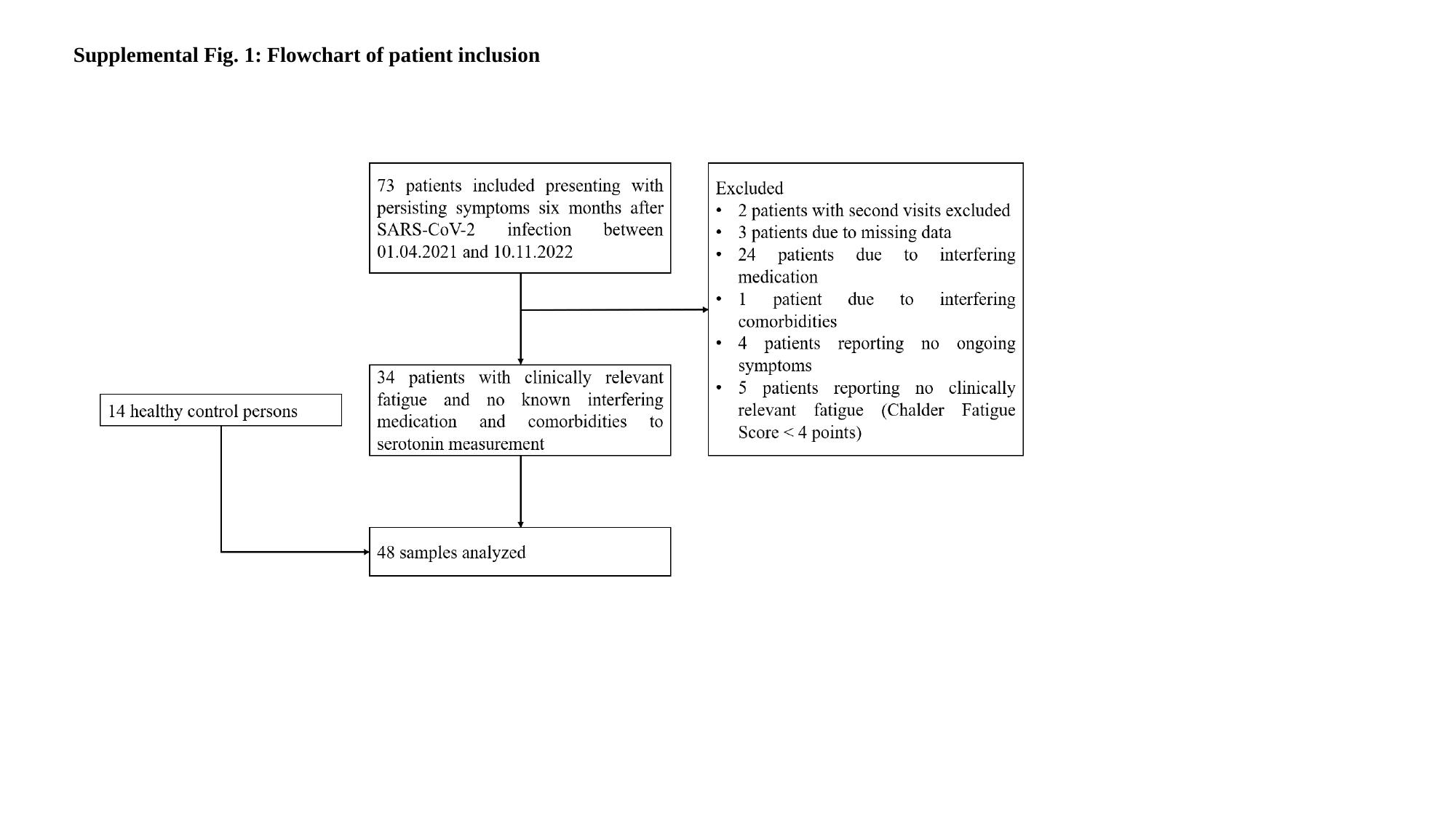

Supplemental Fig. 1: Flowchart of patient inclusion

Supplement: Supplementary file 1 — Supplementary file1 (PPTX 141 KB) [file 15010_2024_2397_MOESM1_ESM.pptx]
